# Deep Reinforcement Learning Based Trajectory Planning Under Uncertain Constraints

**DOI:** 10.3389/fnbot.2022.883562

**Published:** 2022-05-02

**Authors:** Lienhung Chen, Zhongliang Jiang, Long Cheng, Alois C. Knoll, Mingchuan Zhou

**Affiliations:** ^1^Department of Computer Science, Technische Universität München, Munich, Germany; ^2^College of Computer Science and Artificial Intelligence, Wenzhou University, Wenzhou, China; ^3^College of Biosystems Engineering and Food Science, Zhejiang University, Hangzhou, China

**Keywords:** reinforcement learning, neural networks, trajectory planning, collision avoidance, uncertain environment, robotics

## Abstract

With the advance in algorithms, deep reinforcement learning (DRL) offers solutions to trajectory planning under uncertain environments. Different from traditional trajectory planning which requires lots of effort to tackle complicated high-dimensional problems, the recently proposed DRL enables the robot manipulator to autonomously learn and discover optimal trajectory planning by interacting with the environment. In this article, we present state-of-the-art DRL-based collision-avoidance trajectory planning for uncertain environments such as a safe human coexistent environment. Since the robot manipulator operates in high dimensional continuous state-action spaces, model-free, policy gradient-based soft actor-critic (SAC), and deep deterministic policy gradient (DDPG) framework are adapted to our scenario for comparison. In order to assess our proposal, we simulate a 7-DOF Panda (Franka Emika) robot manipulator in the PyBullet physics engine and then evaluate its trajectory planning with reward, loss, safe rate, and accuracy. Finally, our final report shows the effectiveness of state-of-the-art DRL algorithms for trajectory planning under uncertain environments with zero collision after 5,000 episodes of training.

## 1. Introduction

Multi-Degree-of-Freedom (Multi-DOF) robotic arm is widely used in a variety of automation scenarios, including the automotive industry, equipment fabrication, food industry, health care, and agriculture. In the past, Multi-DOF robotic arms usually operated in isolated, structured environments, and tasks that need to adapt to actual conditions are often done by humans. Human-Robot Collaboration (HRC) combines the flexibility of humans and the efficiency of robots, making manufacturing more flexible and productive (Vysocky and Novak, 2016). However, it is a challenge for traditional motion planning algorithms to define a safe, collision-free HRC system, since all its parameters are established based on a specific environment which makes it difficult to adapt new workspace. Probability Road Map (PRM) and Rapidly-exploring Random Tree (RRT) for instance, are not suitable for dynamics environments, since they require higher real-time performance of algorithms to deal with dynamic obstacles, i.e., they need to construct a real-time mapping of obstacles in the configuration space so as to plan a collision-free path, which is very computationally expensive (Adiyatov and Varol, 2017; Kurosu et al., 2017; Wei and Ren, 2018; Wittmann et al., 2020; Jiang et al., 2021; Liu et al., 2021). Another common approach, potential field (PF), has less computation and better real-time control compared to PRM and RRT, however, it often gets stuck in the local minimum, and has limited performance when the obstacles are in the vicinity of the target (Flacco et al., 2012; Lu et al., 2018, 2021; Xu et al., 2018; Melchiorre et al., 2019; Zhou et al., 2019). Therefore, finding an efficient, safe, and flexible motion planning algorithm is required. Reinforcement learning (RL) paves an alternative way to solve these challenges, especially in many high-dimensional tasks or games, RL can exhibit its outperformance. In DeepMind, it is even thought to be enough to reach general AI (Shanahan et al., 2020).

Recently, deep RL, which leverages neural networks as function approximation, has been proven its effectiveness in many different kinds of high complexity of robotic control tasks. Joshi et al. (2020) shows multiple RGB images with double-deep Q-learning can reach over 80% success rate in different grasping tasks without training on a large dataset. In Gu et al. (2017), the robot learns to complete a door opening task with DDPG and Normalized Advantage Function algorithm (NAF) with only a few hours of training. Another example shown in Haarnoja et al. (2018a) with soft Q-learning a robot can learn how to stack Lego blocks together within 2 h of policy training. Therefore, we carry out an idea, using the DRL-based method to tackle complex trajectory planning under uncertain environments. However, deep RL still faces some challenges: (1) defining an appropriate reward function is not straightforward, especially dealing with high dimensional problems, it is easy to obtain the result we incentivize instead of what we intended. (2) In simple tasks, normally an RL agent can discover an optimal policy in a short period, however when encountering complex tasks it may take a few million training steps to achieve the desired result. (3) It is hard to prevent an RL agent from overfitting, to overcome this problem an agent should be trained on a large distribution of environments, but it's very computationally expensive.

In this article, collision-free trajectory planning under uncertain environments is tackled with state-of-the-art DRL algorithms. Since the robotic systems are high dimensional and the state, action space is continuous, model-free Deep Neural Networks (DNN) approaches for Q- and policy-function approximations are used, which has shown its effectiveness in Amarjyoti (2017). Moreover, we expect our approaches to be more suitable for continuous and stochastic environments as well as to have higher sample efficiency and stability, we leverage a combined version, actor-critic based network, which updates the policy network for better action choices also updates the value network for more precise evaluation on policy at each step (Sutton and Barto, 2018). The primary contributions of this paper are summarized as follows:

- Construct an appropriate dense reward function that includes distance-to-goal reward and distance between obstacles reward, and the weight between both rewards is tuned by comparing the performance across different random seeds, in order to make sure the robot manipulator can follow the goal as long as possible, while also avoid collision with dynamic obstacles.
- Build an uncertain environment in a physics engine to simulate a human coexistent environment and apply state-of-the-art DRL algorithms to 7-DOF robots. Then compare the accuracy, safe rate, and reward of two model-free, policy gradient-based algorithms, SAC, and DDPG.
- To further improve learning efficiency and stability, the state space of goal and obstacles are set to relative position and velocity instead of absolute so that the RL agent can learn the correlation between the end-effector and obstacles as well as the goal, as shown in Section 4.

The rest of the article is structured as follows. Section 2 discusses the study related to the traditional trajectory planning method. Section 3 presents our method and its workflow. Section 4 demonstrates our experiment setup and evaluation of our proposed approaches. Section 5 gives the conclusion of this article and future study.

## 2. Related Study

There are currently some possible solutions to trajectory planning and obstacle avoidance. With its probability completeness and exploration efficiency, the RRT has been widely applied in Multi-DOF manipulator's collision-free trajectory planning. Adiyatov and Varol (2017) introduced RRT Fixed Nodes Dynamic (RRT*FND), with the procedures of Reconnect and Regrow in the RRT*FND algorithm, the manipulator can repair the path with an average of 300 ms when encountering an invalid path caused by a dynamic obstacle. Wei and Ren (2018) proposed an improved RRT algorithm, called Smoothly RRT (S-RRT), to generate a smoother path and more stable motion when avoiding obstacles which have shown better exploring speed and exploring efficiency than Basic-RRT and Bi-RRT. In a dual-arm robot pick-and-place environment, Kurosu et al. (2017) regard one of the robot arms as a dynamic obstacle, leveraging the RRT algorithm to effectively avoid collision with another arm during pick-and-place tasks. Although RRT-based algorithm has shown its robustness in either dynamic or static obstacles avoidance, constantly, and randomly moving obstacles avoidance, still requires more research.

Another common approach for collision avoidance trajectory planning is the artificial potential field (APF). This method leverages the PF force of attraction for reaching the goal and repulsion for avoiding obstacles. Xu et al. (2018) leverage a similar algorithm to APF, called velocity potential field (VPF), to avoid collision with a static/dynamic obstacle and a collaborative robot arm. They use the velocity of the robot instead of the distance in APF to avoid suffering from local minima problems when attractive and repulsive forces/velocities confront each other on the same line. Flacco et al. (2012) leverage a simple version of APF, a repulsive vector, generated by the distance to estimate obstacles velocity for collision avoidance. Melchiorre et al. (2019) also leverage the repulsive vector with the distance calculated from the point cloud and have also shown its effectiveness in avoiding collision with static/dynamic obstacles. However, the PF has limited performance when encountering two obstacles that are placed too close to each other. For example, if the goal is in-between or behind two close obstacles, the robot will neglect the goal and turn away.

The other approach is PRM, which takes random samples from the configuration space of the robot and finds a collision-free path between the start and goal nodes. Liu et al. (2021) proposed a Grid-Local PRM that combined a mapping model, sampling strategies, lazy collision detection, and a single local detection method. This proposed method can implement for dynamic path planning for static/dynamic obstacle avoidance. Wittmann et al. (2020) introduce the Obstacle-related Sampling Rejection Probabilistic Roadmap planner (ORSR-PRM). They leverage PRM for trajectory planning and PF for obstacle avoidance in real-time. Similar to the PF method, the probability of generating nodes in between narrow passages is very small, and hence, no path will be planned through the gap.

To overcome the above problem in traditional path planning methods, we proposed another method. Instead of finding a path in configuration space or tackling complicated optimization problems, we leverage the model-free DRL method that allowed the manipulator to autonomously learn optimal collision-free trajectory planning in an uncertain environment.

## 3. Methods

The four essential parts of RL are policy, reward function, value function, and model of the environment. With the idea that an intelligent agent should learn to take a sequence of actions that will lead to maximizing cumulative rewards interacting with the environment. Hence, the agent should *exploit* what it has experienced in order to obtain rewards, but also *explore* in order to make better action decisions in the future (Sutton and Barto, 2018). The Basic RL problem is modeled as the Markov decision process (MDP) with elements *S*_*t*_, *A*_*t*_, *P*(*S*_*t*+1_|*S*_*t*_, *A*_*t*_), γ, *R*(*S*_*t*+1_|*S*_*t*_, *A*_*t*_), where *t* represents timestep, *S*_*t*_ and *S*_*t*+1_ represent the current state and next state, respectively, *A*_*t*_ stands for the current action, *P*(*S*_*t*+1_|*S*_*t*_, *A*_*t*_) stands for the transition probability of being in *S*_*t*+1_ when taking action *A*_*t*_ in the current state *S*_*t*_, and γ ∈ [0, 1) represents discount factor which determines the importance of future rewards, *R*(*S*_*t*+1_|*S*_*t*_, *A*_*t*_) represents the immediate reward received after transitioning from the current state *S*_*t*_ to the next state *S*_*t*+1_, due to the taken action *A*_*t*_. In MDP, we assume that the transition probability (or the probability of moving to the next state *S*_*t*+1_) depends on the current state *S*_*t*_ and the decision action *A*_*t*_. But given *S*_*t*_ and *A*_*t*_, it is conditionally independent of all previous states and actions.

### 3.1. Deep Reinforcement Learning

In the case of complex systems such as robotic systems, the explicit model of the dynamics in the environment associated with MDP, i.e., transition probability function, is often not available or difficult to define. Therefore, a model-free-based method is required. Q-learning is one of the most important breakthroughs in RL also known as the off-policy Temporal Difference (TD) control algorithm, defined by


(1) $$\begin{array}{l} \begin{array}{c} Q\left(S_{t},A_{t}\right)\leftarrow Q\left(S_{t},A_{t}\right)\\ \;+\alpha \left[R_{t+1}+\gamma \;\underset{a}{\max}Q\left(S_{t+1},a\right)-Q\left(S_{t},A_{t}\right)\right], \end{array} \end{array}$$

where *S*_*t*_ and *A*_*t*_ are state and action in timestep *t*, respectively. α stands for learning rate. *R*_*t*+1_ is the obtained immediate reward due to the taken action *A*_*t*_. *a* represents the action that has a maximum *Q*-value from the state *S*_*t*+1_. The optimal action-value function can be directly approximated by the learned action-value function *Q*, which dramatically simplified the analysis of the algorithm and has been proven for convergence (Sutton and Barto, 2018).

In traditional Q-learning, we utilize Q-table to help track states, actions, and corresponding expected rewards. However, for continuous action and state-space such as robotic systems, it is infeasible to build up a large table. Therefore, we need a function approximation for the action-value function *Q* and a DNN is one of the efficient and easy techniques to approximate a non-linear function. However, RL with DNN is pretty unstable, the weights of the network can oscillate or diverge due to the high correlation between actions and states. To overcome this issue, we need to leverage two important techniques, *Experience Replay*, and *Target Network*. By Experience Replay, the agent's experience at each time step will be stored in replay memory as the tuple (*S*_*t*_, *A*_*t*_, *R*_*t*+1_, *S*_*t*+1_), and when the replay memory size is equal to or bigger than a mini-batch size, we then uniformly sample the memory randomly for a mini-batch of experience and use this to learn off-policy, in order to break the correlation (Lin, 1992). Moreover, to make training more stable, a target network is used for calculating the estimate of optimal future value $\underset{a}{\max}Q\left(S_{t+1},a\right)$ in the Bellman equation, and hence, the loss function can be defined as


(2) $$\begin{array}{l} L(\theta )=\{\left[R_{t+1}+\gamma \underset{a}{\max}Q(S_{t+1},a;\theta_{target})\right]\\ \;{-Q(S_{t},A_{t};\theta_{prediction})\}}^{2}, \end{array}$$

where θ_*prediction*_ are prediction network's weights updated in every iteration, whereas θ_*target*_ are the target network's weights, which are not trained but periodically synchronized with the parameters of the prediction Q-network.

### 3.2. The Proposed DRL-Based Trajectory Planning for Uncertain Environments

In this section, we define the setup for the DRL framework, such as the state space S, the action space A, and the reward function.

#### 3.2.1. State Space

**Figure 1:**
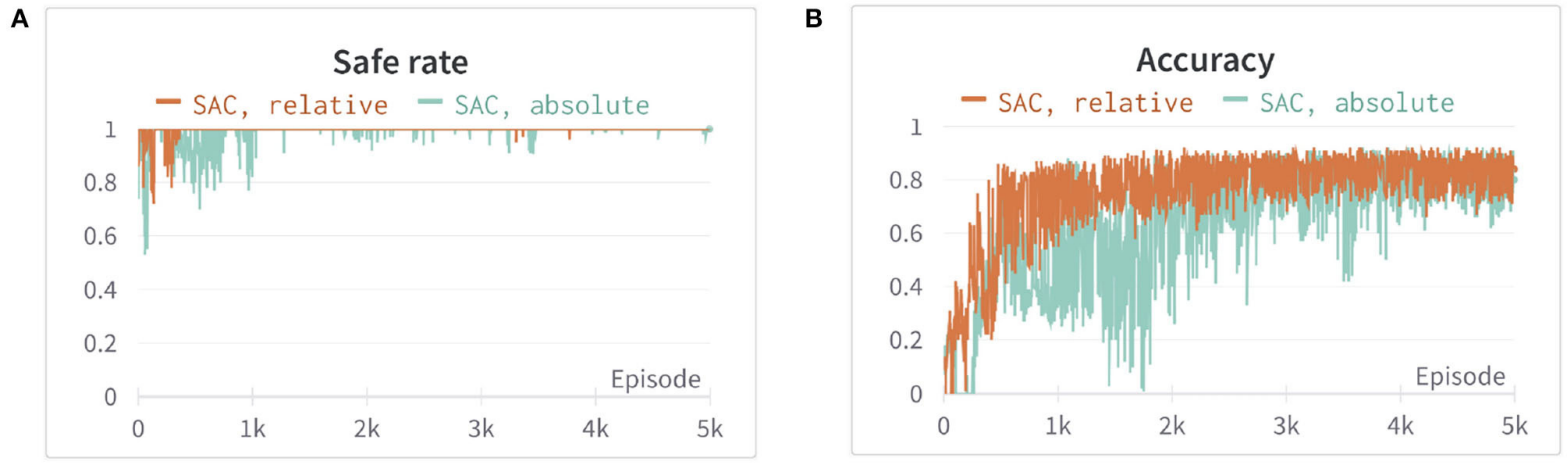
The comparison of two different observation spaces set up in the first environment. Both are using soft actor-critic (SAC) and with the same hyperparameters setting. The orange line is the result using relative position and velocity in observation space, whereas the blue line is using position and velocity. **(A)** The safe rate (defined in Section 4.2) of different observation spaces. **(B)** The accuracy (defined in Section 4.2) of different observation spaces. Each episode corresponds to 100 time steps.

In our experiment the robot manipulator, we used is 7-DOF, therefore, if we set joint positions and velocity as the observations, the learning efficiency of the agent is quite low (Henderson et al., 2018) or may even be unable to find the optimal trajectory. To overcome this issue, we instead use the end-effector position *p*_*e*_ and velocity $\dot{p_{e}}$ then calculate inverse kinematic (IK) to control joint position. Moreover, we use relative position and velocity to the end-effector instead of obstacles or the goal position $\bar{p_{o}}$/$\bar{p_{t}}$ and velocity $\dot{\bar{p_{o}}}$ / $\dot{\bar{p_{t}}}$, which has shown faster convergence and higher stability in Figure 1. The above information is assumed known and obtained from the sensor. Furthermore, to increase learning efficiency, we constrain the manipulator in a specific workspace, and hence, the robot will only explore its reachable area and the area with the goal nearby. The State-space S is hence defined as


(3) $$\begin{array}{l} S=\left\{p_{e},\;\dot{p_{e}},\;\bar{p_{t}},\;\dot{\bar{p_{t}}},\;\bar{p_{o}},\;\dot{\bar{p_{o}}}\right\}. \end{array}$$

#### 3.2.2. Action Space

As we mentioned in section A, we set the end-effector position as the observation for better learning efficiency. Therefore, we can reduce the dimension of actions from seven dimensions to three dimensions with fixed orientations. The action space A is defined as


(4) $$\begin{array}{l} A=\left\{\Delta x,\;\Delta y,\;\Delta z\right\}, \end{array}$$

where Δ*x*,Δ*y*,Δ*z* are bounded between −0.1 and 0.1 such that we can avoid sudden movements of the robotic arm in every time step due to excessive output of the action.

#### 3.2.3. Reward Function

**Figure 2:**
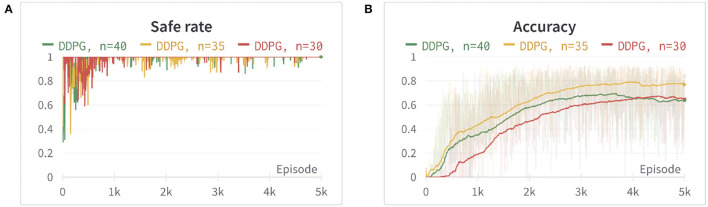
The comparison between different power (*n*) of the exponential decay function. **(A)** The safe rate (defined in Section 4.2) of three different power (*n*) of the exponential decay function. **(B)** The accuracy (defined in Section 4.2) of three different power (*n*) of the exponential decay using an exponential moving average for better visualization. The two figures show that *n* = 35 has better learning efficiency, safe rate, and accuracy.

**Figure 3:**
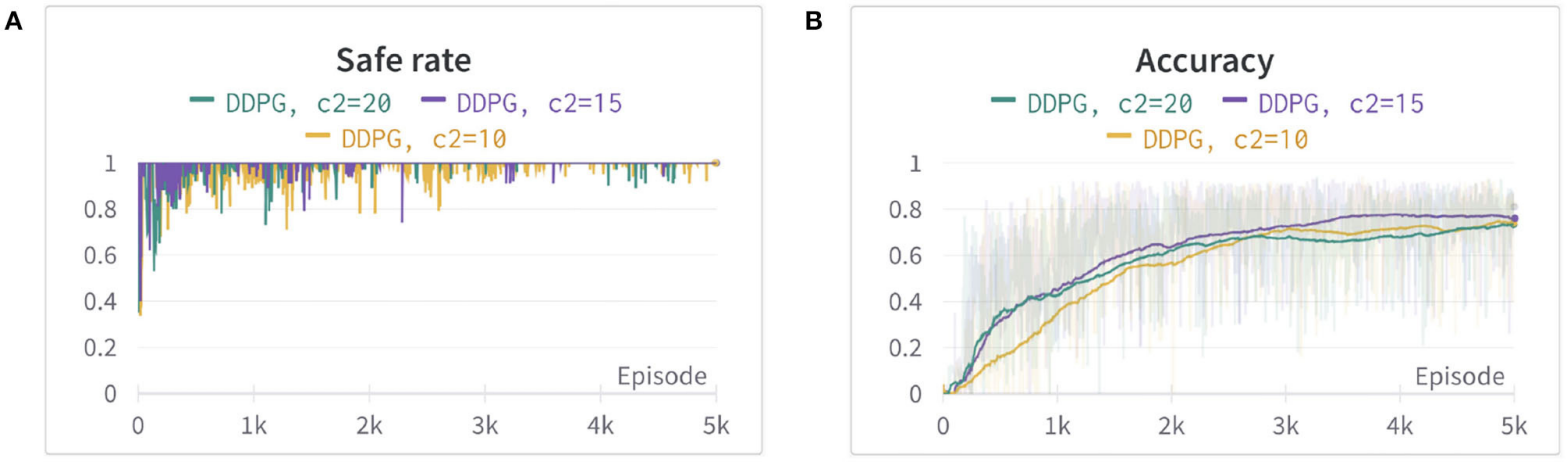
The comparison between different weights of reward *R*_*O*_. **(A)** The safe rate (defined in Section 4.2) of three different weights of reward *R*_*O*_. **(B)** The accuracy (defined in Section 4.2) of three different weights of reward *R*_*O*_ using an exponential moving average for better visualization. The two figures show that *c*2 = 15 has better learning efficiency, safe rate, and accuracy.

**Figure 4:**
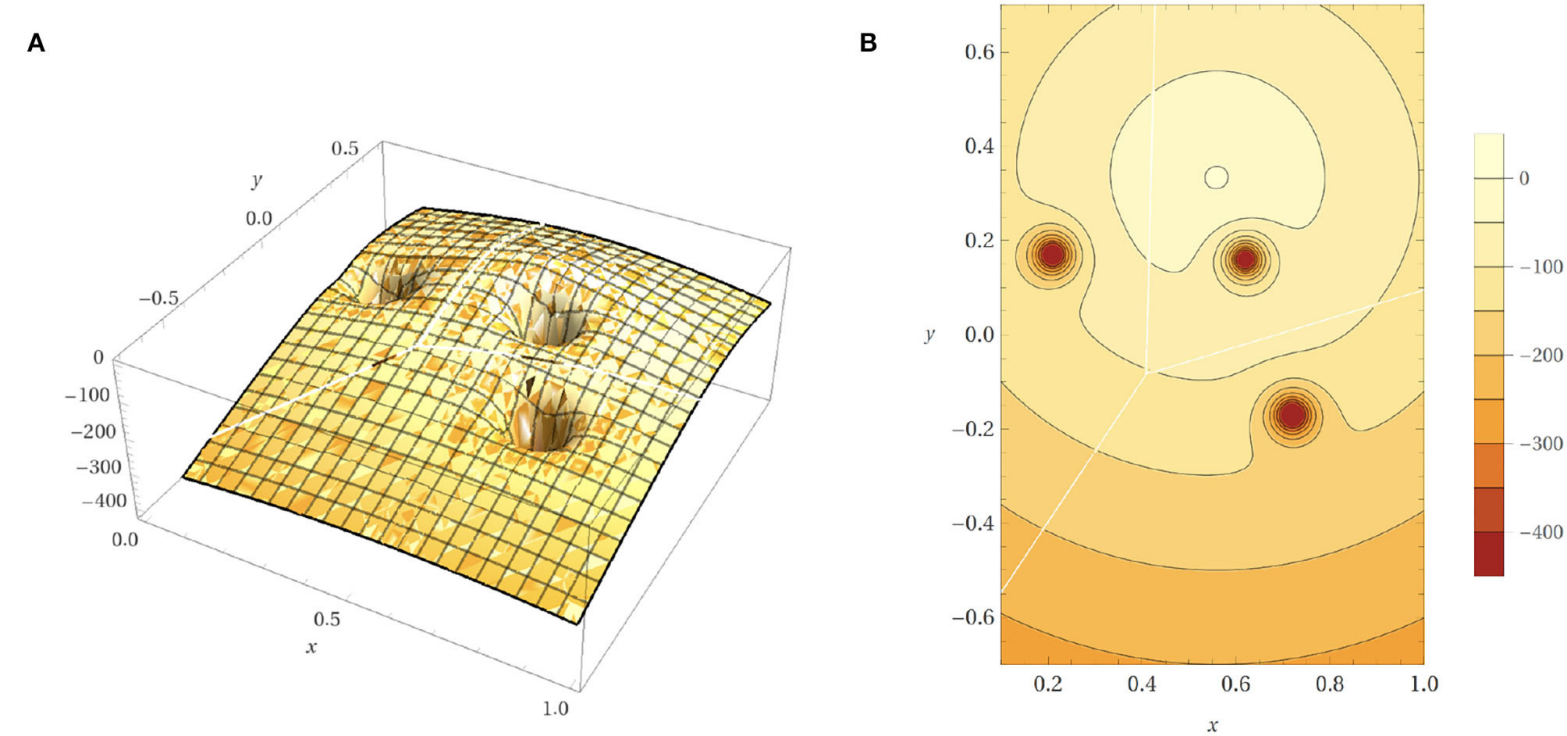
Reward function on the planar section of the workspace. **(A)** The 3D plot of the reward function. **(B)** Contour plot of reward function.

**Figure 5:**
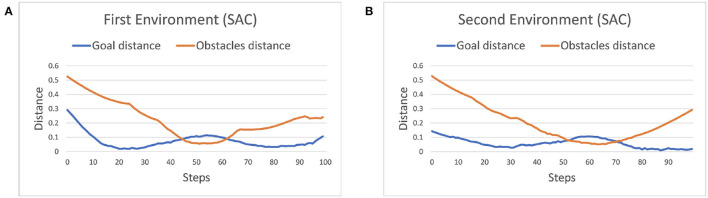
The behavior of the manipulator with respect to the distance(m) in one of the episodes (100-time steps). **(A)** Distance between robot and obstacles as well as the target (first scenario). **(B)** Distance between robot and obstacles as well as the target (second scenario).

The reward function mixes reward variables into a single output value and provides feedback for an agent to learn what we incentive. In our case, we expect the robot to follow the goal as long as possible while avoiding dynamic obstacles. Therefore, we define the reward function by a weighted sum of two terms: First, the distance between the end-effector and the goal. Second, the closest distance between the robot manipulator and obstacles. Moreover, we use negative a reward over a positive so that the robot will try its best to avoid penalties and, hence, can learn as quickly as possible. The reward function is defined as


(5) $$\begin{array}{l} R=-c_{1}R_{T}-c_{2}R_{O}, \end{array}$$

where *R*_*T*_ is the reward obtained from the distance between the end-effector and the goal using Euclidean distance (*m*),


(6) $$\begin{array}{l} R_{T}=\frac{1}{2}d_{T}^{2}, \end{array}$$

where *d*_*T*_ is the Euclidean distance between the end-effector and the goal. The reward *R*_*O*_ is obtained from the closest distance (*m*) between the robot manipulator and obstacles,


(7) $$\begin{array}{l} R_{O}={\left(\frac{a}{a+d_{O}}\right)}^{n}, \end{array}$$

where *d*_*O*_ is the closest distance from the obstacles computed by PyBullet. *a* is set to 1, in order to avoid the denominator equaling zero when a collision happens. The power of exponential decay function *n* = 35 and the weights *c*2 = 15 are determined by using trial and error. We set *c*1 as a fixed value of 500 and tune the parameters *n* and *c*2 by evaluating the safe rate, accuracy, and learning efficiency, as shown in Figures 2, 3 (Since DDPG is more sensitive to parameters, we use DDPG for comparison; Haarnoja et al., 2018b). In order to show our reward function has a maximum, we plot our reward function on the planar section of the workspace, as shown in Figure 4. Moreover, since the dynamic/static goal and dynamic obstacles are on the same x-y plane, it can be demonstrated in 3D space instead of 4D for better visualization. As it can be observed from Figure 4, the reward decreases as the robot's end-effector moves toward obstacles and increases as it moves toward the goal, and when the end-effector reaches the goal point, the reward is maximum. The behavior of the robot manipulator in two environments is also shown in Figure 5. The distance between the end-effector and goal diminishes as the robot approaches the goal and when obstacles are close to the body of the robot, the robot backs off until obstacles move away from the manipulator.

#### 3.2.4. Deep Deterministic Policy Gradient

Deep deterministic policy gradient (DDPG), introduced in Lillicrap et al. (2015), is an actor-critic, model-free algorithm based on the deterministic policy gradient that can operate over continuous action spaces. Traditionally, in policy gradient-based algorithms the policy function is always stochastic, i.e., it is modeled as a probability distribution over actions given the current state. In DDPG, the policy function is instead modeled as a deterministic decision. However, this may lead to a low exploration issue, and hence, they add additive noise to the deterministic action to explore the environment, which is represented as:


(8) $$\begin{array}{l} \mu^{\prime }\left(S_{t}\right)=\mu \left(S_{t}|\theta_{t}^{\mu }\right)+N, \end{array}$$

where $\mu {\left(S_{t}\right)}^{\prime }$ and N are the exploration policy and additive noise, here, use an Ornstein-Uhlenbeck process. $\mu \left(S_{t}|\theta_{t}^{\mu }\right)$ and $\theta_{t}^{\mu }$ are the output action and parameters of the actor-network. Moreover, the traditional target networks are updated with the parameters of the trained networks every couple of thousand steps, which may cause big differences between the two updates. Therefore, they introduced a *soft target update*, which is actually better to make the target networks slowly track the trained networks, by updating their parameters after each update of the trained network using a sliding average for both the actor and the critic:


(9) $$\begin{array}{l} \theta^{\prime }\leftarrow \tau \theta +\left(1-\tau \right)\theta^{\prime },\;with\;\tau \ll 1, \end{array}$$

where θ and θ′ represent parameters for the actor- or critic-network and the target actor- or target critic-network. τ is the target smoothing coefficient. The Experience Replay mentioned in Section A is also used here to store past trajectories and provides samples of them to perform gradient updates for better learning efficiency. The detailed pseudo algorithm of DDPG-based trajectory planning is shown in Algorithm 1.

**Algorithm 1:** DDPG-based trajectory planning.

**Input:** batch size: *B*, target smoothing coefficient τ, discount factor: γ, number of training episode: *M*, timesteps of each episode: *T*
Randomly initialize Q network *Q*(*S, A*|θ^*Q*^) and policy network μ(*S*|θ^μ^) with weights θ^*Q*^ and θ^μ^
Initialize target Q network *Q*′ and target policy network μ′ with weights θ^*Q*^′ ← θ^*Q*^, θ^μ^′ ← θ^μ^
Initialize replay buffer *D* ← ϕ
**Input:** A set of observation state $S=\left\{p_{e},\dot{p_{e}},\bar{p_{t}},\dot{\bar{p_{t}}},\bar{p_{o}},\dot{\bar{p_{o}}}\right\}$
**Output:** A set of optimal policy A={Δx,Δy,Δz}
**for** episode = 1, *M* **do**
Initialize a random noise N for action exploration
Receive initial observation state *S*_1_
**for** t = 1, *T* **do**
Select action $A_{t}=\mu \left(S_{t}|\theta^{\mu }\right)+N_{t}$ according to the
current policy and exploration noise
Execute action *A*_*t*_ and observe reward *R*_*t*_ and
observe new state *S*_*t*+1_
Store transition (*S*_*t*_, *A*_*t*_, *R*_*t*_, *S*_*t*+1_) in *D*
Sample a random minibatch of *B* transitions
(*S*_*i*_, *A*_*i*_, *R*_*i*_, *S*_*i*+1_) from *D*
Set $y_{i}=R_{i}+\gamma Q^{\prime }\left[S_{i+1},\mu^{\prime }\left(S_{i+1}|\theta^{\mu^{\prime }}\right)|\theta^{Q^{\prime }}\right]$
Update critic by minimizing the loss:
$L=\frac{1}{B}\underset{i}{\sum }{\left[y_{i}-Q\left(S_{i},A_{i}|\theta^{Q}\right)\right]}^{2}$
Update the actor policy using the sampled policy
gradient: $\nabla_{\theta^{\mu }}J\approx$
$\frac{1}{B}\underset{i}{\sum }\nabla_{A}Q\left(S,A|\theta^{Q}\right){|}_{S=S_{i},A=\mu \left(S_{i}\right)}\nabla_{\theta^{\mu }}\mu \left(S|\theta^{\mu }\right){|}_{S_{i}}$
Update the target networks:
$\theta^{Q^{\prime }}\leftarrow \tau \theta^{Q}+\left(1-\tau \right)\theta^{Q^{\prime }}$
$\theta^{\mu^{\prime }}\leftarrow \tau \theta^{\mu }+\left(1-\tau \right)\theta^{\mu^{\prime }}$
**end for**
**end for**

#### 3.2.5. Soft Actor-Critic

Similar to DDPG, soft actor-critic (SAC) introduced in Haarnoja et al. (2018b), is also an actor-critic, model-free algorithm that can operate over continuous action spaces, but is based on the stochastic policy by maximizing the expected reward of the actor while maximizing entropy, i.e., achieve the goal while acting as randomly as possible. Hence, the general maximum entropy can be represented as:


(10) $$J(\pi )=\sum_{t=0}^{T}{𝔼}_{(S_{t},A_{t})\sim \rho_{\pi }}[r(S_{t},A_{t})+\alpha ℋ(\pi (\cdot |S_{t}))],$$

where ρ_π_ and $H\left(\pi \left(\cdot |s_{t}\right)\right)$ are the policy and the entropy. α is the temperature parameter for determining the relative importance of the entropy term against the reward, and hence controls the stochasticity of the optimal policy. Since SAC is an off-policy algorithm, the Experience Replay is also used to improve the learning efficiency. Moreover, in order to decrease the changes between two updates, the soft target update technique in Equation (4) is also applied. In Haarnoja et al. (2018b), they also compare with some other on-policy DRL algorithms, such as TRPO (Schulman et al., 2015), PPO (Schulman et al., 2017), or A3C (Mnih et al., 2016), and has shown its higher sample efficiency. Compare with DDPG, it also has lower hyperparameter sensitivity and higher stability, which is also our case shown in Section 4. The detailed pseudo algorithm of SAC-based trajectory planning is shown in Algorithm 2.

**Algorithm 2:** SAC-based trajectory planning.

**Input:** batch size: *B*, target smoothing coefficient τ, discount factor: γ, number of training episode: *M*, timesteps of each episode: *T*
Randomly initialize Q network *Q*_1_, *Q*_2_, policy network π and value network *V* with weights θ_1_, θ_1_, ϕ and ψ.
Initialize target Q networks $Q_{1}^{\prime }$ and $Q_{2}^{\prime }$, target value network *V*′ with weights $\theta_{1}^{\prime }\leftarrow \theta$, $\theta_{2}^{\prime }\leftarrow \theta$ and ψ′ ← ψ
Initialize replay buffer *D*
**Input:** A set of observation state $S=\left\{p_{e},\dot{p_{e}},\bar{p_{t}},\dot{\bar{p_{t}}},\bar{p_{o}},\dot{\bar{p_{o}}}\right\}$
**Output:** A set of optimal policy A={Δx,Δy,Δz}
**for** episode = 1, *M* **do**
Receive initial observation state *S*_1_
**for** t = 1, *T* **do**
Select action *A*_*t*_ ~ π_ϕ_(*A*_*t*_|*S*_*t*_)
Execute action *A*_*t*_ and observe reward *R*_*t*_ and
observe new state *S*_*t*+1_
Store transition (*S*_*t*_, *A*_*t*_, *R*_*t*_, *S*_*t*+1_) in *D*
Sample a random minibatch of *B* transitions
(*S*_*i*_, *A*_*i*_, *R*_*i*_, *S*_*i*+1_) from *D*
Update V by minimizing the mean squared error:
$\nabla_{\psi }J_{V}(\psi )=\frac{1}{B}\sum_{i}\nabla_{\psi }V_{\psi }(S_{i})[V_{\psi }(S_{i})$
$-\min_{j=1,2}Q_{{\theta^{\prime }}_{j}}(S_{i},A_{i})+log\pi_{\phi }(A_{i}|S_{i})]$
Update Q by minimizing the soft Bellman residual:
$\nabla_{\theta_{1,2}}J_{Q}\left(\theta_{1,2}\right)=\nabla_{\theta_{1,2}}\frac{1}{B}\underset{i}{\sum }$
$\{{\left[Q_{\theta_{1}}(S_{i},A_{i})-\alpha R(S_{i},A_{i})-\gamma V_{\psi^{\prime }}(S_{i+1})\right]}^{2}-$
${\left[Q_{\theta_{2}}(S_{i},A_{i})-\alpha R(S_{i},A_{i})-\gamma V_{\psi^{\prime }}(S_{i+1})\right]}^{2}\}$
Update π by minimizing the expected KL-
divergence:
$\nabla_{\phi }J_{\pi }\left(\phi \right)=\nabla_{\phi }\frac{1}{B}\sum_{i}[\log\pi_{\phi }\left(A_{i}|S_{i}\right)-$
$\min_{j=1,2}\;Q_{\theta_{j}^{\prime }}\left(S_{i},A_{i}\right)]$
Update the target value networks:
$\psi^{\prime }\leftarrow \tau \psi +\left(1-\tau \right)\psi^{\prime }$
**end for**
**end for**

## 4. Experiment and Results

In this section, we show that DDPG and SAC can learn optimal trajectory planning for dynamic obstacles collision avoidance. For the evaluation, we compare two different DRL algorithms with safe rate, accuracy, and reward in two different environments.

### 4.1. Environment Setup

**Figure 6:**
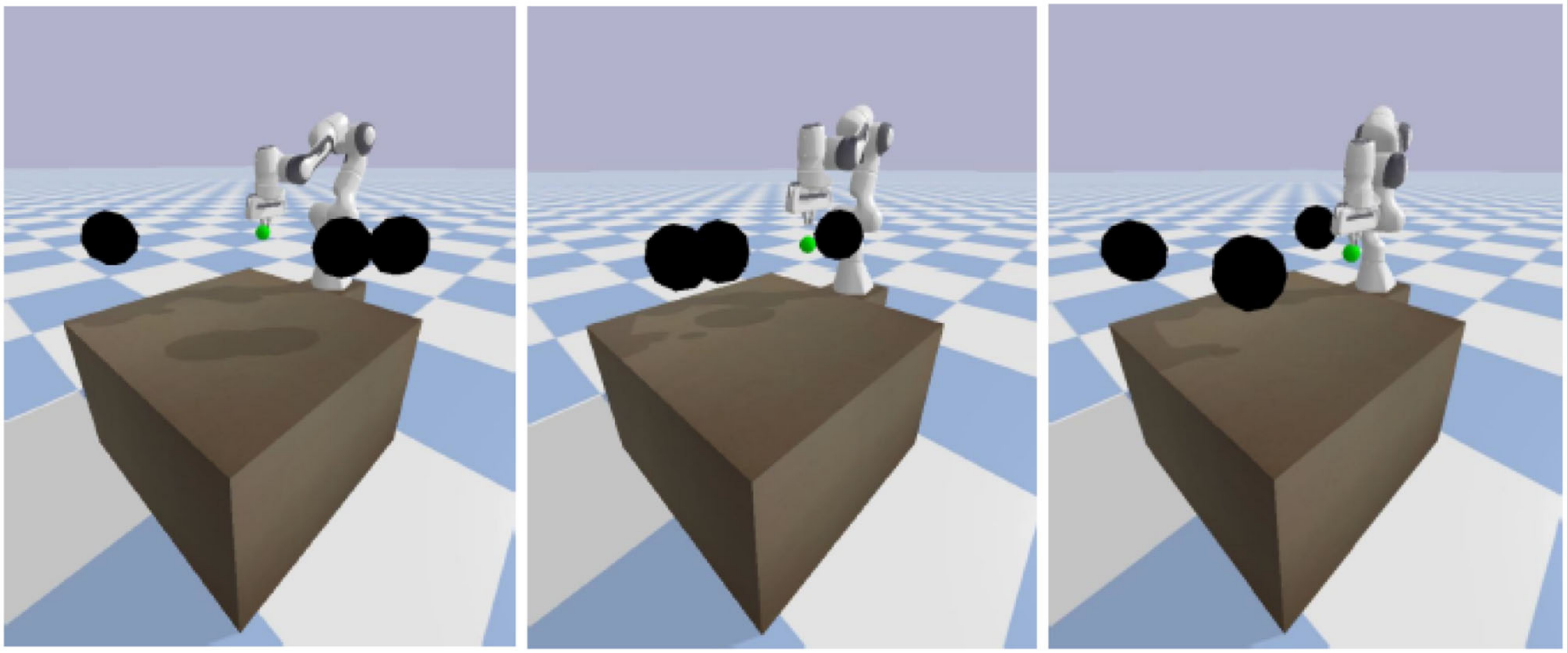
From left to right, first, the robot learns to reach the goal. Second, avoid collisions with dynamic obstacles. Third, keep reaching the goal.

In our experiment, we applied the proposed collision avoidance DDPG and SAC algorithm on a 7-DOF manipulator (Panda from Franka Emika) simulated in a PyBullet physics engine and leveraged the RL toolkit Gym. The environment setup contains a manipulator, a table, a green sphere goal, and three black sphere obstacles, as shown in Figure 6. Moreover, we constructed two environments for the evaluation, either a static goal and dynamic obstacles or a dynamic goal and dynamic obstacles. Besides, in order to make sure our model can learn under uncertainty, the starting positions of the goal and three obstacles are uniformly and randomly sampled in a specific range, and the goal moving areas are constrained in the robot's reachable area.

### 4.2. Evaluation

We evaluate different DRL algorithms in two defined scenarios with the safe rate, accuracy, reward, and loss. Each episode corresponds to 100 time steps, i.e., the robot has to reach the goal and avoid collision within 100 time steps. The safe rate represents the number of time steps without collision divided by 100 time steps (one episode),


(11) $$\begin{array}{l} \text{Safe rate}=\frac{\text{number of timesteps without collision}}{100\text{ timesteps}}. \end{array}$$

The accuracy stands for a success rate of keeping a distance between the target within 0.05 *m* while far away from obstacles, and 0.12 *m* while avoiding collision with obstacles (the radius of obstacles is 0.1 *m*), therefore, it is not possible to obtain 100% of accuracy, since it also includes time steps from the rest position to the target. The accuracy is set as,


(12) $$\begin{array}{l} \text{Accuracy}=\frac{\text{number of timesteps that succeed}}{100\text{ timesteps}}. \end{array}$$

The two algorithms' performances were evaluated in two different environments using the same set of parameters respectively, such as learning rate, number of hidden layers, target smoothing coefficient. The environment settings considered for the experiments are (1) dynamic goal and dynamic obstacles: the starting position of both goal and obstacles are uniformly and randomly sampled in a specific range for each episode, and moving with constant speed. (2) fixed goal and dynamic obstacles: the obstacles remain in the same setting as the first one, but with a fixed goal sampled randomly for each episode.

**Figure 7:**
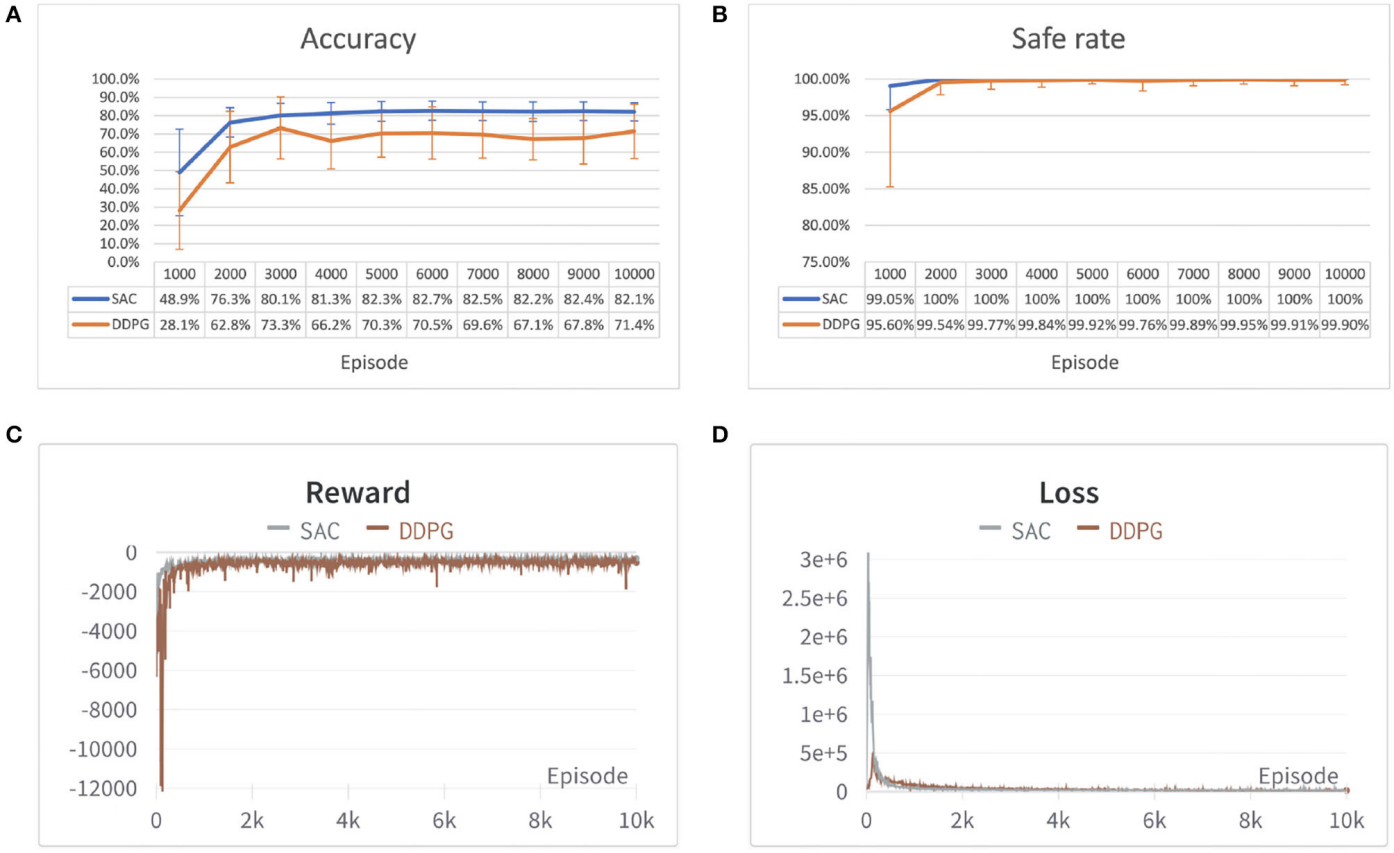
Performance comparison of SAC and Deep Deterministic Policy Gradient (DDPG) algorithm in the first environment. **(A)** Accuracy of different algorithms shown in error bar line graph. **(B)** A safe rate of different algorithms in the error bar line graph. **(C)** The cumulative reward for each episode. **(D)** Loss for each episode. Each episode corresponds to 100 time steps.

**Figure 8:**
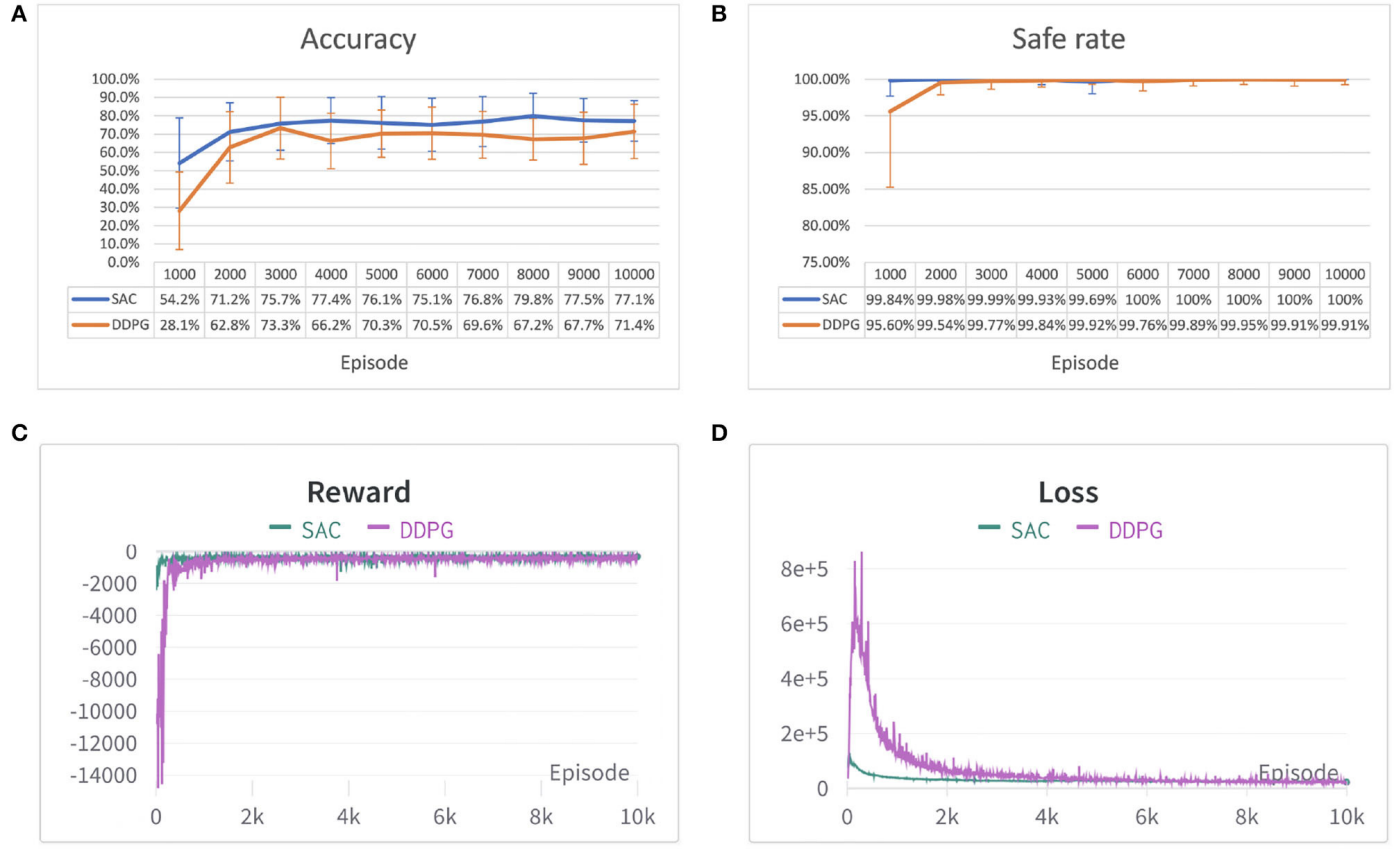
Performance comparison of SAC and DDPG algorithm in the second environment. **(A)** Accuracy of different algorithms shown in error bar line graph. **(B)** A safe rate of different algorithms in the error bar line graph. **(C)** The cumulative reward for each episode. **(D)** Loss for each episode. Each episode corresponds to 100 time steps.

In each experiment, the safe rate, accuracy, reward, and loss per episode have been traced during the training process and compared after 10,000 episodes of 100 time steps each. The result of the two environments is shown in Figures 7, 8. From both Figures 7C,D, 8C,D it can be observed that both algorithms' cumulative reward converges to their maximum value, and the losses do not have significant reductions, i.e., the robot has learned a stable optimal trajectory planning under an uncertain environment. Moreover, both Figures 7A, 8A show that SAC performs much more consistently, efficiently, and higher accuracy, whereas deterministic policy-based DDPG exhibits high variability between episodes and less stable. Furthermore, both Figures 7B, 8B demonstrate that SAC can learn a collision free trajectory with 100% of safe rate within 6,000 episodes, while DDPG still cannot guarantee to reach a 100% of safe rate within 10,000 episodes. Similar results are also corroborated in Gu et al. (2016) and Haarnoja et al. (2018b). The reason for that is because the interplay between the deterministic actor-network and the Q-function makes DDPG unstable and sensitive to hyperparameters, especially for complex and high-dimensional tasks, however, DDPG still shows its effectiveness in both scenarios. Overall, the performance of the stochastic policy-based SAC is more stable and consistent when dealing with complex tasks.

## 5. Conclusion and Future Study

In this article, we presented two state-of-the-art off-policy DRL approaches that can be used to discover optimal trajectory planning under an uncertain environment. Especially, stochastic policy-based SAC can achieve an average of 82% of accuracy in the first scenario and 79% in the second, moreover, with lower variability between episodes and zero collision after 5,000 episodes. The results show the clear potential of the proposed approaches in the application of an uncertain environment, such as HRC scenarios. The future study will transfer the trained model from a simulation environment to real physical robotic manipulators and transfer the learning skill from simulation to the real environment with visual sensing.

## Data Availability Statement

The original contributions presented in the study are included in the article/supplementary material, further inquiries can be directed to the corresponding author/s.

## Author Contributions

LChen implemented the code and draft the manuscript. ZJ assisted to implement the code and discussed the manuscript. LCheng assisted to implement the code and discussed the manuscript. AK guided the research and discussed the results. MZ guided the research, implemented parts of code, and revised the manuscript.

## Funding

This study was supported by the National Natural Science Foundation of China (Grant Numbers: 32101626 and 61902442) and ZJU 100 Young Talent Program. This study was supported in part by the German Research Foundation (DFG) and in part by the Technical University of Munich (TUM) in the framework of the Open Access Publishing Program.

## Conflict of Interest

The authors declare that the research was conducted in the absence of any commercial or financial relationships that could be construed as a potential conflict of interest.

## Publisher's Note

All claims expressed in this article are solely those of the authors and do not necessarily represent those of their affiliated organizations, or those of the publisher, the editors and the reviewers. Any product that may be evaluated in this article, or claim that may be made by its manufacturer, is not guaranteed or endorsed by the publisher.
